# Double gate operation of metal nanodot array based single electron device

**DOI:** 10.1038/s41598-022-15734-1

**Published:** 2022-07-06

**Authors:** Takayuki Gyakushi, Ikuma Amano, Atsushi Tsurumaki-Fukuchi, Masashi Arita, Yasuo Takahashi

**Affiliations:** Graduate School of Information Science and Technology, Hokkaido University, Sapporo, 060-0814 Japan

**Keywords:** Electronic devices, Electrical and electronic engineering

## Abstract

Multidot single-electron devices (SEDs) can enable new types of computing technologies, such as those that are reconfigurable and reservoir-computing. A self-assembled metal nanodot array film that is attached to multiple gates is a candidate for use in such SEDs for achieving high functionality. However, the single-electron properties of such a film have not yet been investigated in conjunction with optimally controlled multiple gates because of the structural complexity of incorporating many nanodots. In this study, Fe nanodot-array-based double-gate SEDs were fabricated by vacuum deposition, and their single-electron properties (modulated by the top- and bottom-gate voltages; *V*_T_ and *V*_B_, respectively) were investigated. The phase of the Coulomb blockade oscillation systematically shifted with *V*_T_, indicating that the charge state of the single dot was controlled by both the gate voltages despite the metallic random multidot structure. This result demonstrates that the Coulomb blockade oscillation (originating from the dot in the multidot array) can be modulated by the two gates. The top and bottom gates affected the electronic state of the dot unevenly owing to the geometrical effect caused by the following: (1) vertically asymmetric dot shape and (2) variation of the dot size (including the surrounding dots). This is a characteristic feature of a nanodot array that uses self-assembled metal dots; for example, prepared by vacuum deposition. Such variations derived from a randomly distributed nanodot array will be useful in enhancing the functionality of multidot devices.

## Introduction

Recently, new types of computing technologies that use nanodot devices have been an active field of research; such as quantum^1–4^, reconfigurable^5–7^, and reservoir-computing^8,9^. A single-electron device (SED) can enable these technologies because of its high functionality and ultralow power consumption^10–13^. A SED exhibits a unique characteristic, known as the Coulomb blockade oscillation, with which the drain current (*I*_D_) is periodically modulated by the gate voltage (*V*_G_). This Coulomb blockade oscillation is caused by the charging effect of a nanodot (also known as the single-electron island) contained in the SED and is not observed in a conventional metal–oxide–semiconductor transistor. In previous studies, efforts were made to arrange a single dot between the SED electrodes, and the basic characteristics of the device^14–18^ were evaluated for applications associated with conventional logic circuits^11,12,19–24^. Using Si-based SEDs, the inverter operation^20^, half-sum and carry-out operation^21^, and multivalue memories^22^ have been demonstrated thus far.

Considering the single-nanodot SED known as the single-electron transistor (SET), which contains one nanodot between the source and drain electrodes, multiple SETs must be connected via wiring to form logic circuits. In this case, the one-by-one electron tunneling that occurs in SETs requires time for the wires to charge and the operation is slow. Additionally, the size variability of the nanodots induces the scattering of the SET characteristics. To overcome these difficulties, a nanodot-array-based SED that contains multiple nanodots without wiring was proposed, and its functionality was demonstrated under multigate operation^5,6,25–28^. For example, a flexible logic gate operation was enabled by using an Si nanodot array SED, indicating that nonuniform capacitive coupling between the dots and gate electrodes is a key factor for achieving logic operations^25–27^.

Besides nanodot fabrication methods that use lithography as reported in previous work, dispersion of chemically synthesized nanodots or self-assembled nanodot growth by thin-film deposition can be used to form nanodot array devices^5,29–35^. In these cases, the array comprises randomly distributed nanodots and numerous conduction paths with different properties must form. Although single-electron tunneling properties might be difficult to detect in such complex systems, Coulomb blockade properties are frequently detected;^29–38^ and the modulation that is caused by the gate voltage is evident and straightforward in some cases^32,35,37,38^. To determine the potential of these complex array SEDs, further investigations using multigate operation are required. However, single-electron properties controlled by multiple gate voltages, which are important fundamentals for functional SEDs, have not been studied yet. In a randomly distributed nanodot array, nonuniform capacitive coupling between the dots and gate is expected due to the variation of the dot shape and size of the surrounding dots. In accordance with numerical simulations in Ref.^25^, such variations of the gate capacitance are hypothesized to provide complex Coulomb blockade oscillations characteristics, which might enable a multidot SED to achieve logic operations.

In this study, an Fe nanodot single layer embedded in MgF_2_ and sandwiched between two gate electrodes was used for SED fabrication; and modulation of *I*_D_ against the top- and back-gate voltages (*V*_T_ and *V*_B_, respectively) was systematically measured using SEDs with straightforward Coulomb blockade oscillation. It was demonstrated that the straightforward Coulomb blockade oscillations were controlled by *V*_T_ and *V*_B_, despite the metal and randomly distributed multidot structure. It was also found that the Coulomb blockade oscillations were unevenly responsive to both *V*_T_ and *V*_B_. These results indicate the application potential of a complex array SED in functional devices, including logic gates. The unevenness of the gate operations depends on the thickness of the gate insulators, the dot shape asymmetry (hemispheric rather than spheric), and the arrangement of the surrounding nanodots.

## Methods

**Figure 1:**
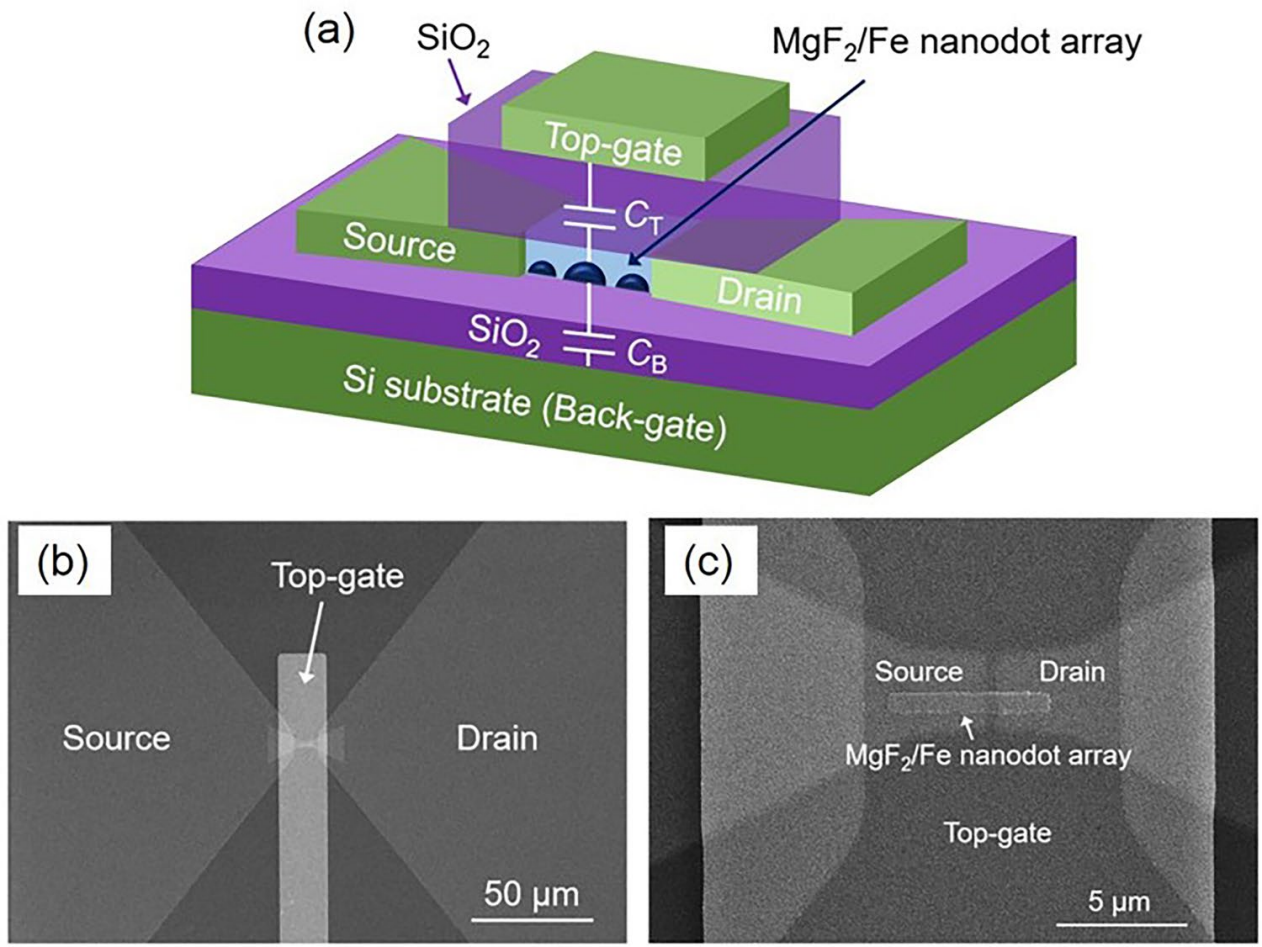
(**a**) Schematic and (**b**) and (**c**) scanning electron microscopy images (plan view) of the fabricated Fe nanodot array device.

Figure 1 shows a schematic, and scanning electron microscopy images, of the fabricated Fe nanodot device. The source and drain electrodes (Au/Cr) with a gap length *L* of 50–400 nm were formed on a thermally oxidized SiO_2_ (200 nm)/Si substrate. Afterward, an MgF_2_ (45 nm)/Fe (film thickness *t*_Fe_ = 1.8–2.9 nm) layer was formed between the electrodes at room temperature by using electron-beam deposition (base pressure < 10^−7^ Pa). In this *t*_Fe_ range, dispersed Fe nanodots were formed owing to surface migration and aggregation of Fe atoms on SiO_2_. Finally, the gate-insulating SiO_2_ (300 nm) layer was prepared on the MgF_2_ layer via sputtering, followed by top-gate electrode (Au/Cr) formation. Details of the device fabrication were given in a previous study^37^. The drain current between the source and drain electrodes (*I*_D_) was measured using a semiconductor parameter analyzer (Agilent 4156C) by applying the voltages *V*_T_ and *V*_B_ to the Au/Cr top-gate and Si (substrate) back-gate electrodes, respectively, in a closed-cycle cryogenic probe station at a sample stage temperature *T* = 8 K.

## Results and discussion

**Figure 2:**
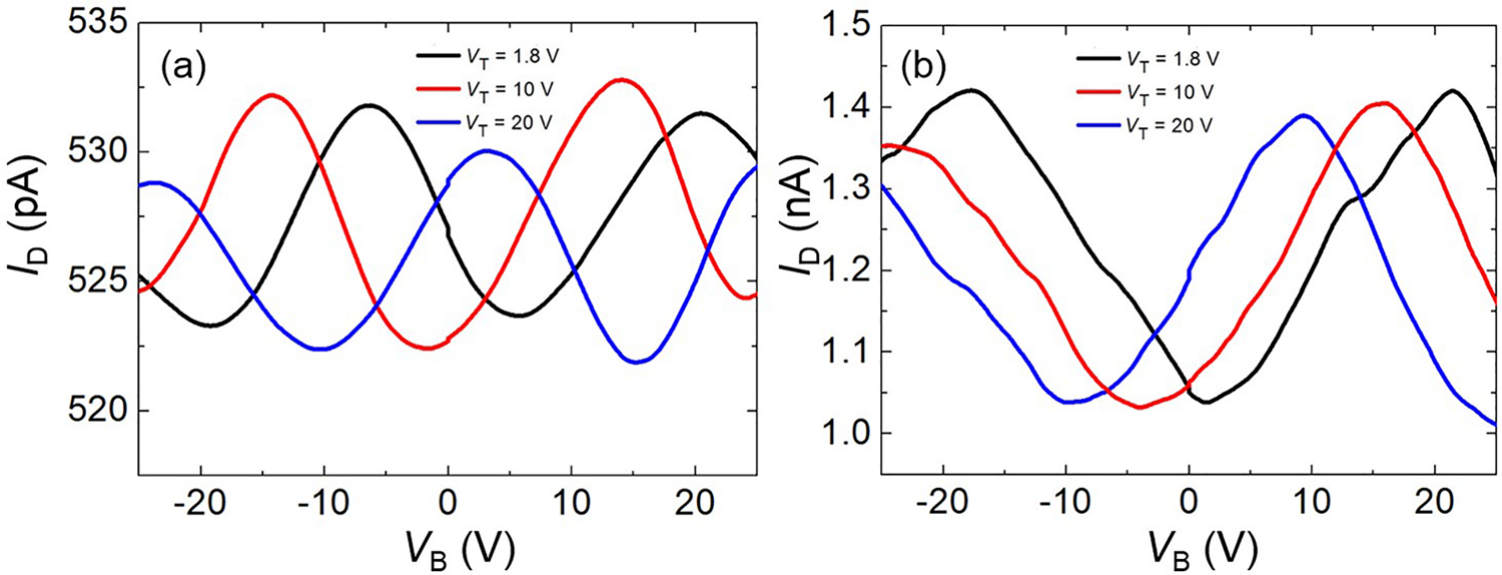
Current oscillations originating from a single dot measured as a function of the back-gate voltage *V*_B_ for the following: (**a**) Device A (*t*_Fe_ = 2.4 nm and *L* = 50 nm) at drain voltage *V*_D_ = 20 mV and (**b**) Device B (*t*_Fe_ = 2.4 nm and *L* = 400 nm) at *V*_D_ = 5 mV. The top-gate voltage *V*_T_ was constant at 1.8, 10, and 20 V.

Almost all of the fabricated devices exhibited Coulomb blockade oscillations where highly reproducible back-and-forth *I*_D_–*V*_B_ curves with the peak/valley ratio larger than ~ 1.1 were seen. About 95% of the fabricated devices were in this category. Some of them (ca. 10%) showed *I*_D_–*V*_B_ oscillations that originated from a single dot as in our previous study^37^. Typical current oscillation characteristics in this category are demonstrated in Fig. 2 as a function of *V*_B_ for two SEDs, termed Devices A and B in this report. The *V*_D_ value utilized here was sufficiently small such that the Coulomb blockade of the dot was not lifted. A brief discussion of *I*_D_–*V*_D_ characteristics and the charging energy of the dot can be seen in the supplementary information as well as our previous study^37^. In both of the graphs shown in Fig. 2, three *I*_D_–*V*_B_ curves at *V*_T_ = 1.8, 10, and 20 V are superposed. The observed oscillation curves are evident, and they originated from a single dot, as discussed in our previous study^37^. The oscillation period was approximately 27 (Device A) or 37 V (Device B); corresponding to a back-gate capacitance *C*_B_ of 5.9 (Device A) or 4.3 × 10^−3^ aF (Device B), respectively. Device B has a smaller *C*_B_ compared with Device A, the dot that contributes the current oscillation in this device should be smaller than that of Device A. In both devices, the current oscillations were added to the constant-background current components [~ 520 pA (Device A) and ~ 1.0 nA (Device B)], which were attributable to the parallel conductive paths comprising the dots with the Coulomb blockade lifted. Such dots (attributable to the background current) are relatively large and/or exhibit higher tunneling conductance than the quantized value (2*e*^2^/*h*)^37^. By comparing the *I*_D_–*V*_B_ curves with different *V*_T_, a systematic shift in the current peak was identified. With increasing *V*_T_, the peak ca. 20 V for Device A gradually shifted to 14 and 3.3 V (Fig. 2a). A similar tendency was evident for Device B (Fig. 2b), with a peak shift from 22 to 16 V, and then to 9.3 V. These peak shifts toward the negative *V*_B_ direction demonstrate that the current oscillation characteristics can be controlled by both the top and back gates, even in the metal multidot SED, where the change in the nanodot charging state by *V*_T_ can be compensated by *V*_B_ and vice versa.

**Figure 3:**
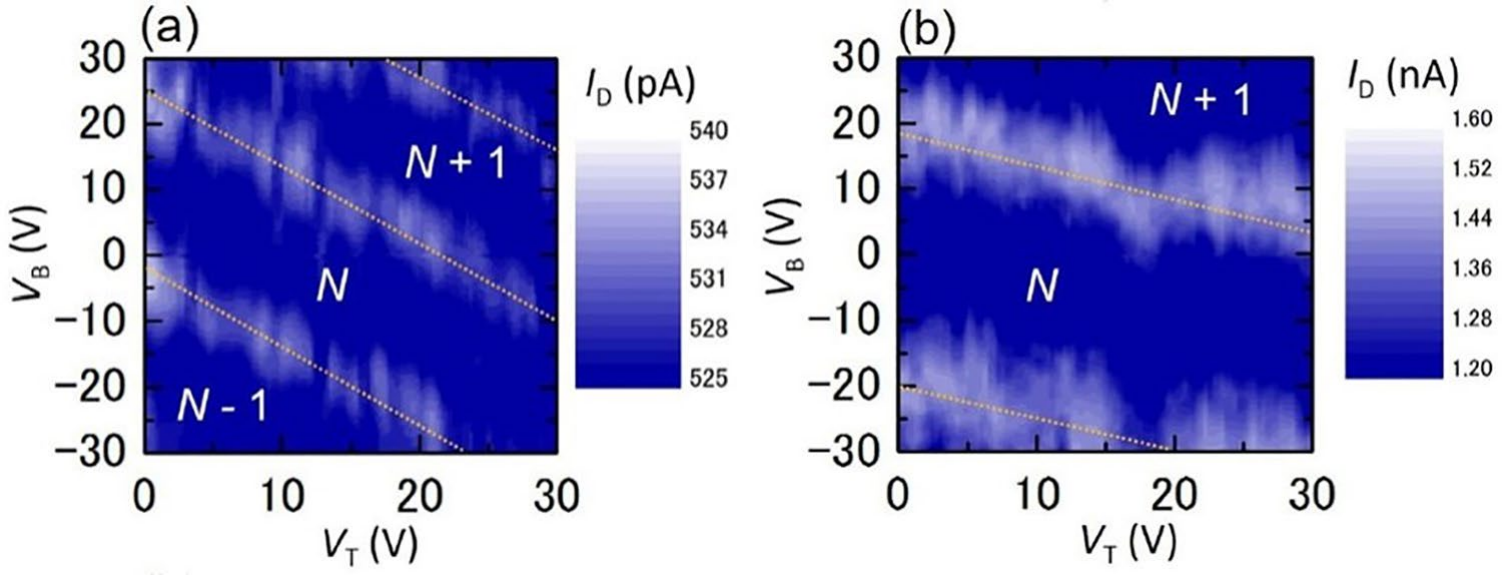
Typical two-dimensional contour-line maps of drain current *I*_D_ as a function of *V*_B_ and *V*_T_. (**a**) Device A at *V*_D_ = 20 mV and (**b**) Device B at *V*_D_ = 5 mV. *N* is the number of electrons in a single dot.

To clearly understand the details of the peak shift as a function of the two gate voltages (i.e., *V*_T_ and *V*_B_), a contour plot of the drain current was used^27^. For this purpose, numerous *I*_D_–*V*_B_ curves were measured using various *V*_T_ values from 0 to 30 V in 300-mV steps, where the *V*_B_ sweep was conducted in the sequences of 0–30, 30 to − 30, and − 30 to 0 V. The current oscillations were well-reproducible for the back-and-forth *V*_B_ sweeps. In addition, the current oscillations were stable over a few days against the peak shift due to the charge offset drift, similar to the findings of a previous study^38^. Figure 3a and b show the data measured from *V*_B_ = 30 to − 30 V as two-dimensional (2D) contour maps of the drain current, corresponding to the stability diagrams of the device. The current peaks shown in bright contrast were systematically shifted. Thus, the phase of the current oscillation can be controlled by using *V*_T_ and *V*_B_. These contour maps were straightforward and periodic, as indicated by the yellow dotted lines, although there was an irregularity in Fig. 3b at *V*_T_ = 17 V, which was caused by charge noise that might be attributable to the effect of satellite nanodots acting as single-electron traps^37^. These characteristics confirm that the major current oscillation originated from a single dot^37^. Controllability of the charge state of a single dot by a double-gate was confirmed despite the multidot structure.

The results shown in Figs. 2 and 3 are well-known phenomena for multigate SETs with a single dot configuration^28,39,40^. However, manifestation of such a phenomenon in metal nanodot arrays is unprecedented and important because of the corresponding suggestion that SEDs comprising randomly dispersed metal multidots can operate as, for example, two-input logic-gate devices.

Via careful analysis of these data, characteristic features of the double-gate SED operation of the self-assembled nanodot system were clarified. For the devices investigated in this study, the top and back gates were capacitively coupled to the SED, and the current peak shift in accordance with the *V*_B_ and *V*_T_ follows Eq. (1):

1 $$ C_{{\text{B}}} V_{{\text{B}}} + C_{{\text{T}}} V_{{\text{T}}} = const, $$

where *C*_B_ and *C*_T_ are the capacitances between the single dot and back/top gates, respectively. When *V*_T_ changes by Δ*V*_T_, the peak shift in the current oscillation (Δ*V*_B_) is given by Eq. (2):^39^

2 $$ \Delta V_{{\text{B}}} = - \left( {C_{{\text{T}}} /C_{{\text{B}}} } \right)\Delta V_{{\text{T}}} . $$

**Figure 4:**
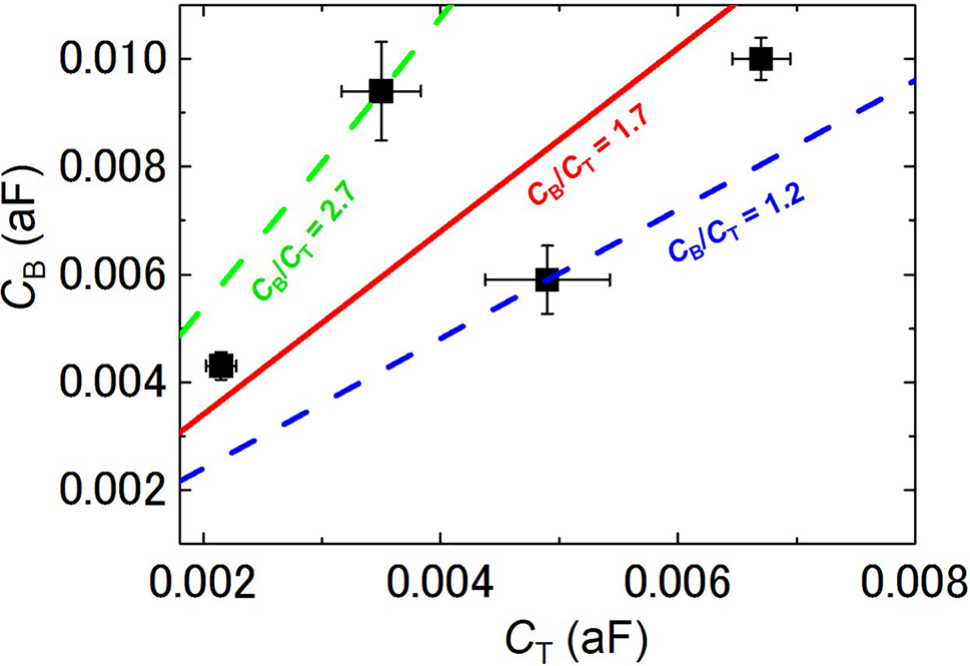
Plots of *C*_B_ vs. *C*_T_ of four devices, indicating simple current oscillation characteristics that originated from a single dot. *C*_B_ of four devices was evaluated by the period of the current peak line. However, *C*_T_ of four devices was evaluated by *C*_B_ and the slope of the current peak line. *C*_B_ includes the fluctuations that were derived from reading errors of the period of the current peak line, which are shown by the vertical error bars. The horizontal error bars represent the fluctuations of *C*_T_ that originated from the fluctuations of *C*_B_. There was a positive correlation between *C*_B_ and *C*_T_. The slope of the dotted line corresponds to the evaluated *C*_B_/*C*_T_ ~ 1.2 (blue) and ~ 2.7 (green). Although the data approximately follow the relation without considering the shape effect of the nanodot system (red line), clear discrepancy from the red line was identified.

Therefore, the gate capacitance ratio *C*_B_/*C*_T_ of the dot is − Δ*V*_T_/Δ*V*_B_, which was evaluated using the slope of the observed current peak line in the contour map^40–44^. Using Fig. 3a and b, the gate capacitance ratio *C*_B_/*C*_T_ between the single dot and back/top gates was ~ 1.2 (Device A) or ~ 2.0 (Device B). Therefore, using the *C*_B_ previously described, *C*_T_ was ~ 4.9 (Device A) or ~ 2.2 × 10^−3^ aF (Device B). Table 1 and Fig. 4 show these results along with data from two other devices. For the device structure investigated in this study, the back-gate insulator comprised 200-nm-thick SiO_2_ and the top-gate insulator comprised 45-nm-thick MgF_2_ and 300-nm-thick SiO_2_. Assuming a parallel-plate capacitor structure and known bulk dielectric constants (3.8 for SiO_2_ and 5.2 for MgF_2_)^45^, the capacitance ratio *C*_B_/*C*_T_ was ~ 1.7 (Fig. 4). For a total set of devices, the evaluated *C*_B_/*C*_T_ followed this relationship. Furthermore, each *C*_B_/*C*_T_ ratio was between 1.2 and 2.7, indicating a clear discrepancy from this straightforward estimation by using the parallel-plane model (~ 1.7). This discrepancy was not caused by measurement and/or estimation errors, but was an important characteristic of the self-assembled dot array. In the following paragraphs, this is discussed by using a simplified model.

**Table 1:** Averaged values of *C*_B_/*C*_T_, *C*_B_, and *C*_T_ for four devices. *C*_B_/*C*_T_ and *C*_B_ contains the reading error of the slope and period of the current peak line, respectively.

Device	*C*_B_/*C*_T_	*C*_B_ (10^−3^ aF)	*C*_T_ (10^−3^ aF)
A	1.2 ± 0.2	5.9 ± 0.6	4.9 ± 0.5
B	2.0 ± 0.4	4.3 ± 0.3	2.2 ± 0.1
C	1.5 ± 0.2	10 ± 0.4	6.7 ± 0.3
D	2.7 ± 0.8	9.4 ± 0.9	3.5 ± 0.3

*C*_T_ was evaluated by using the values of *C*_B_/*C*_T_ and *C*_B_.

**Figure 5:**
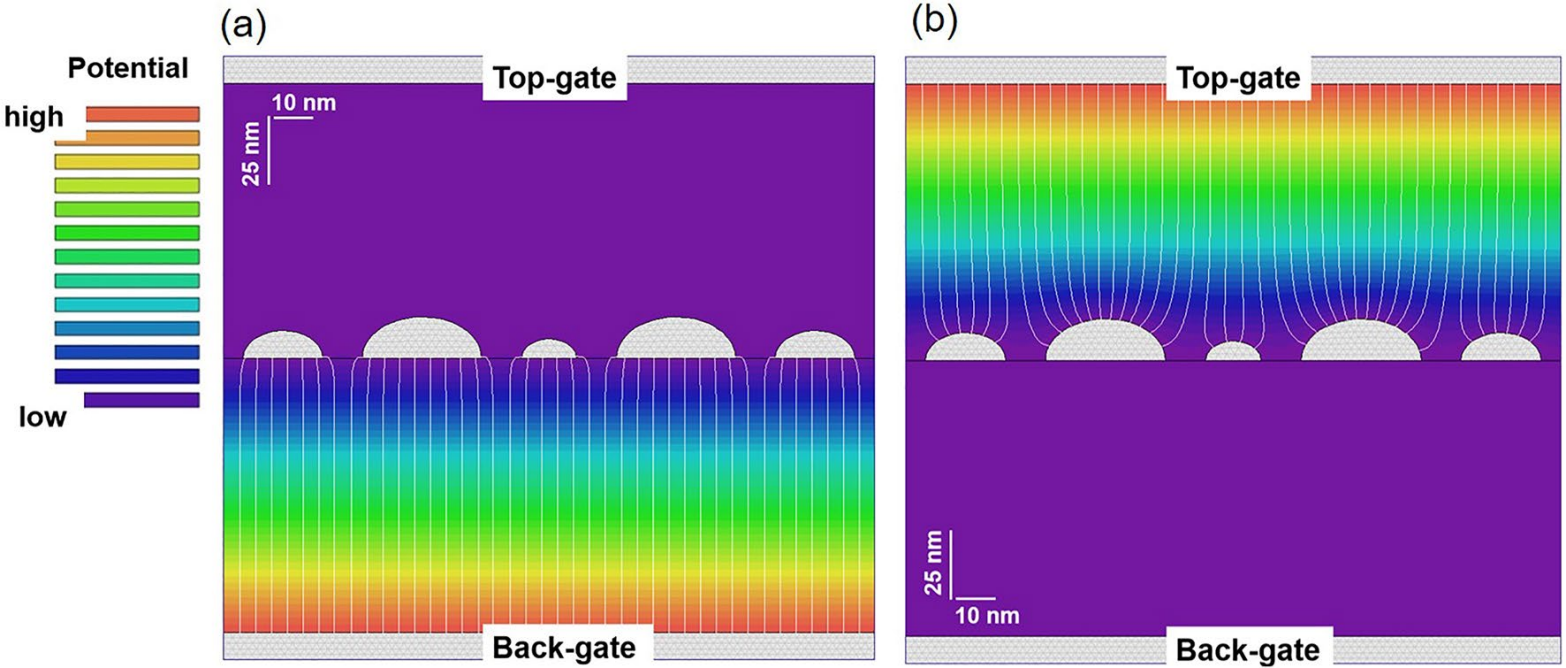
Simulation results of the electric potential (color) and the lines of the electric force (white lines), in which the top or the back electrodes are biased by 1 V. Results for the *LSL* (*L* = large and *S* = small) dot configuration with biased (**a**) back- and (**b**) top-gate. For details of the model, refer to the text.

The nanodot array SED comprised numerous dots with various sizes. Because dots were formed on the substrate plane, the planar arrangement of hemispheric nanodots can be assumed as a model for this discussion. In addition, for straightforward calculations, the model was simplified into parallel-arranged, half-columnar dots with an infinitive axis length. Here, a numerical fit to the experimental data is not the purpose of this simulation. Figure 5 shows an example of the cross-sectional schematic, where the column axis of the dot is perpendicular to the paper surface. The dot is half-circular in this diagram (gray), termed the half-circular dot in the following discussion. By adopting this model, only 2D electric field calculations were required, with few parameters. A compact software (EStat provided by *Advanced Science Laboratory, Inc*.) based on the finite-element method (with an optimized mesh size) was used to solve the Laplace equations for the following: (1) simulating the electric field and (2) evaluating the capacitances (per unit length along the column axis) between the dot and top/back-gate electrodes. Although the model was simplified, the simulation results provided the intrinsic features of the nanodot array. In the next paragraph, the simulation results are explained with details of the model.

Figure 5 shows a typical model for evaluation. Notably, the horizontal and vertical magnifications of this diagram are different from each other. The in-plane diameter of a metallic half-circular dot is 30, 20, or 14 nm; termed *L* (large), *M* (medium), or *S* (small), respectively, throughout this report. The dots were arranged horizontally to form a dot array. In Fig. 5, the dot arrangement is termed *LSL* in the central part and *M* in other regions. The distance between the adjacent dot edges was maintained at 10 nm. The dots were sandwiched between two 100-nm-thick SiO_2_ layers acting as the top- and back-gate insulators. On the surface of these SiO_2_ layers, two metallic gates (gray) were attached. For simulations, one of the gate electrodes was biased by 1 V, whereas the dots and another electrode were grounded. Figure 5 shows simulated potential distributions as color maps; the lines of the electric force are represented by white lines. As described next, a remarkable difference was observed between Fig. 5a for voltage application to the back gate and Fig. 5b for voltage application to the top gate. The electric force lines shown in Fig. 5a were almost parallel, similar to those in a parallel capacitor, except near the dot edges. Thus, *C*_B_ of each dot must be almost proportional to the dot size. Conversely, in Fig. 5b, the electric force lines were strongly curved and those near the central *S* dot were attracted by the adjacent *L* dots. Some of the electric force lines were absorbed by the *L* dots; therefore, *C*_T_ of the central *S* dot became smaller in this dot arrangement, whereas *C*_T_ of the *L* dot became larger. This is because of the geometrical difference between the upper (roundish) and lower (flat) dot surfaces. A dot shape on the nanometer scale can modulate the electric field, changing the charge distribution on the dot surface and the capacitance with the gate electrode. This suggests that the Coulomb blockade oscillation characteristics in a multidot SED comprising a complex dot array can be modulated by the shape and distribution of the dots, including the surrounding dots.

**Figure 6:**
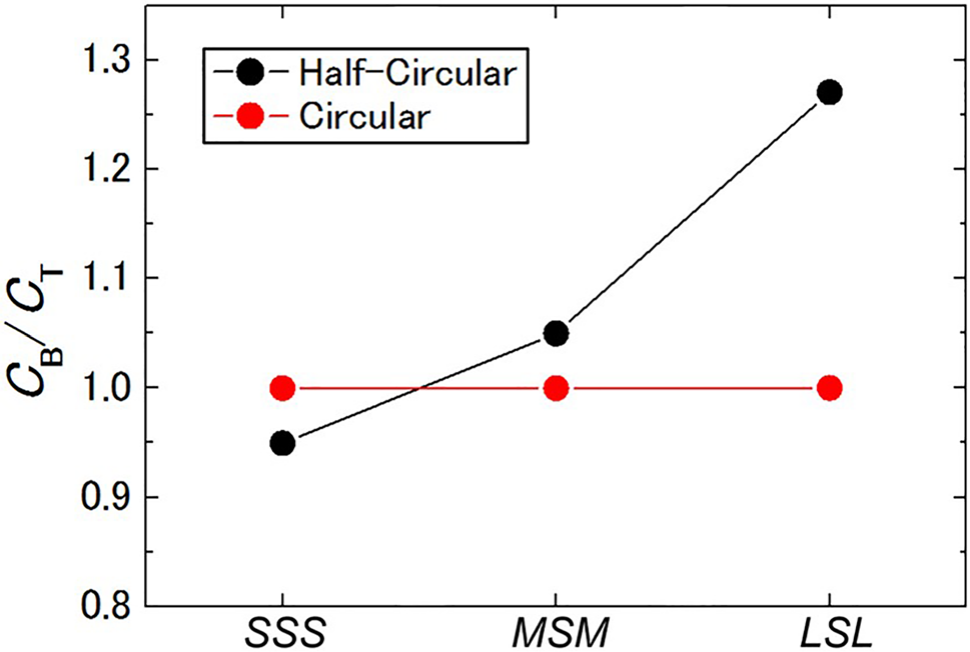
Gate capacitance ratio *C*_B_/*C*_T_ of the central *S* dot for three dot configurations: *SSS*, *MSM*, and *LSL* (*L* = large, *M* = medium, and *S* = small). The *C*_B_/*C*_T_ for circular dots (red) and half-circular dots (black) are presented. The *C*_B_/*C*_T_ value of the circular dots was almost constant at 1.0, regardless of the size of the adjacent dots. Conversely, the value changed from 0.95 to a value of 1.27 for half-circular dots with increasing adjacent dot size.

These simulations were performed for various dot arrangements, and the gate capacitance ratios *C*_B_/*C*_T_ of the central dots were evaluated. Figure 6 shows examples of the *SSS*, *MSM*, and *LSL* arrangements. To check the applicability of the calculations, simulation results of circular dots with the same arrangements were superposed in the graph. Because the dots were symmetric in this case, the *C*_B_/*C*_T_ ratio should be 1.0 in all the cases, reflecting the thickness ratio of the top (100-nm-thick) and bottom (100-nm-thick) insulating layers. The simulation results fit well with this value (the error with the theoretical value was ca. 0.1%). This indicates that the simulation was performed with a sufficient accuracy (number of meshes) to evaluate the *C*_B_/*C*_T_. Considering half-circular dots, the *C*_B_/*C*_T_ value indicates a large change from 0.95 to 1.27, depending on the adjacent dot size. This is because of the nonuniform distribution of the electric field between the top and bottom gates, which was attributable to the geometrical shape effect of the central dot and surrounding dots (Fig. 5) as well as the vertical asymmetric structure of the dots. In the *SSS* arrangement, for example, the roundish shape of the surface that was facing the top gate gathered the electric force lines, rather than the flat surface that was facing the bottom electrode. Therefore, *C*_T_ became larger than *C*_B_ and *C*_B_/*C*_T_ was < 1. For the large adjacent dot, as in the *MSM* arrangement, some electric force lines were attracted by the *M* dots, decreasing the *C*_T_ value; ultimately resulting in *C*_B_/*C*_T_ > 1. Lager adjacent dots in *LSL* strongly attracted the electric force lines from the *S* dot, and the deviation of *C*_B_/*C*_T_ from 1 became large. Similar results were also identified for *M* or *L* as the central dot. Such fundamental discussions by using 2D simulations must also be valid for a three-dimensional dot shape and arrangement. The shape asymmetry of the dots and its positional arrangement resulted in a variation of the *C*_B_/*C*_T_ (and thus the device properties) during double-gate operation.

## Conclusion

A double-gate, Fe nanodot-array-based SED comprising an SiO_2_–Fe–MgF_2_ system was fabricated by using vacuum deposition, and its electric characteristics were investigated. The fabricated Fe nanodot device exhibited current oscillations that originated from a single dot^37^. The charge state of a single dot was controlled by both the top and back gates, even in the multidot structure. This result demonstrates that Coulomb blockade oscillation characteristics that originate from a dot in randomly dispersed multidots can be modulated by two gates. Additionally, the variation of the gate capacitance ratio *C*_B_/*C*_T_ (derived from the randomly distributed and vertically asymmetric nanodot array) was demonstrated. This phenomenon might provide a complex response to the input (gate) voltage. It might be useful to produce flexible logic gates as indicated in previous reports^25–27^, although further comprehensive studies are required in the future. The on/off ratio of the Coulomb blockade oscillations was small because of the many conduction paths that provided the background current. Although this problem is unavoidable in multidot SEDs, the on/off ratio can be improved by using a cascode MOSFET^22,26,46^. The findings of this study will facilitate new applications of metal nanodot arrays.

## Supplementary Information

Supplementary Information.

## Data Availability

The data that support the findings of this study are available from the corresponding author upon reasonable request.
