# Supplementary material for: Double gate operation of metal nanodot array based single electron device
Source: Sci Rep. 2022 Jul 6;12:11446. doi: 10.1038/s41598-022-15734-1 (PMC9259697; doi:10.1038/s41598-022-15734-1)
Supplement: Supplementary file 1 — Supplementary Information. [file 41598_2022_15734_MOESM1_ESM.docx]

Supplementary Information:

**Double Gate Operation of Metal Nanodot Array Based Single Electron Device**

Takayuki Gyakushi,^*^ Ikuma Amano, Atsushi Tsurumaki-Fukuchi, Masashi Arita, and Yasuo Takahashi

*Graduate School of Information Science and Technology, Hokkaido University, Sapporo 060-0814, Japan*

* Corresponding author: gyakushi.takayuki.d8@elms.hokudai.ac.jp

**Typical *I*_D_–*V*_D_ characteristics of devices that exhibited Coulomb blockade oscillations**

Fig. S1(a) shows typical *I*_D_–*V*_D_ characteristic for a device (*t*_Fe_ = 2.6 nm, *L* = 50 nm) that exhibited Coulomb blockade oscillation, which mainly originated from a single dot [Fig. S1(b)]. The *I*_D_–*V*_D_ characteristic was nonlinear because of the Coulomb blockade of the dot around *V*_D_ = 0 V. Although the Coulomb blockade region was not clear, *V*_D_ = 6 mV [arrow in Fig. S1(a)] was sufficiently low to facilitate a condition in which the Coulomb blockade of the dots was not lifted; and thus oscillation was evident in Fig. S1(b). The charging energy of the dot is estimated to be at most a few meV as discussed in our previous study^1^ comparing with the dot diameter. Therefore, the *I*_D_–*V*_D_ characteristic shown in Fig. S1(a) is reasonable.

Figure S2 are the *I*_D_–*V*_D_ characteristics of Devices A and B. Both devices exhibited almost linear *I*_D_–*V*_D_ characteristics in the narrow *V*_D_ scan range ca. 0 V. The scan range of *V*_D_ was within the Coulomb blockade regime, as expected from Fig. S1.


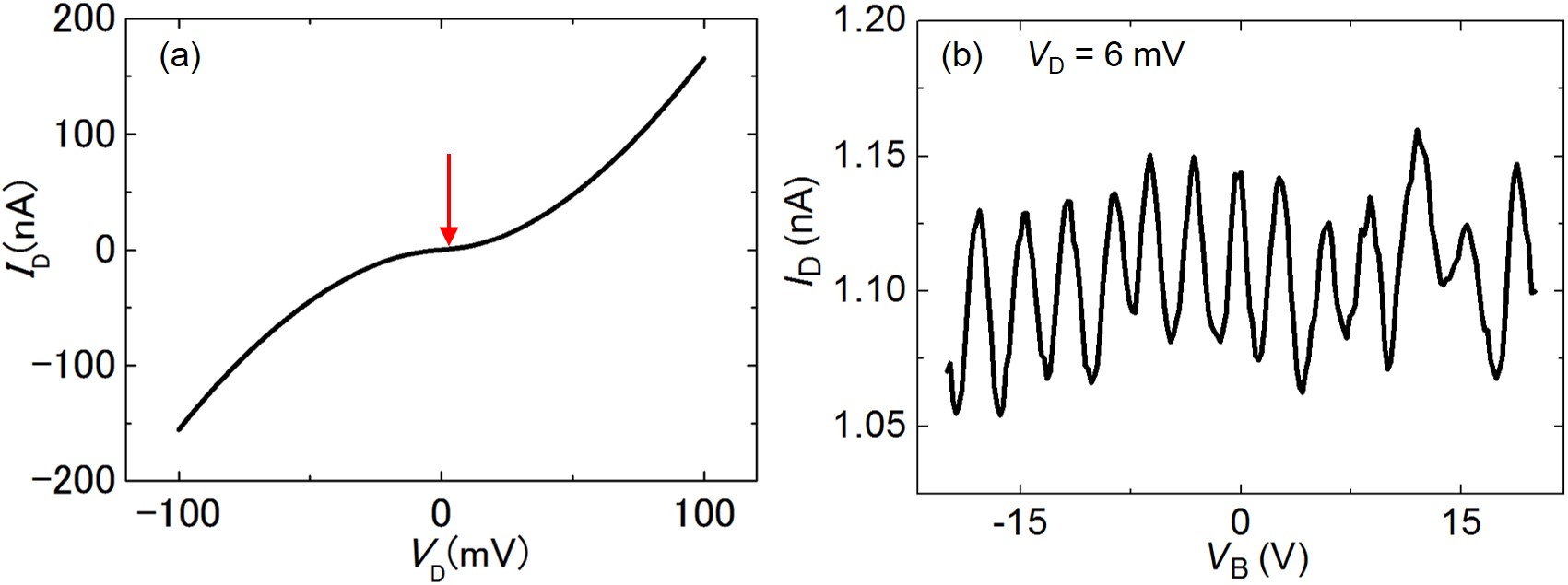


**Figure S1.** (a) Typical *I*_D_–*V*_D_ characteristics, measured in a forward drain voltage scan for the device (*t*_Fe_ = 2.6 nm, *L* = 50 nm). (b) Coulomb blockade oscillation was measured in a forward voltage scan of the back gate at *V*_D_ = 6 mV. The corresponding condition is marked with a red arrow in Fig. S1(a).


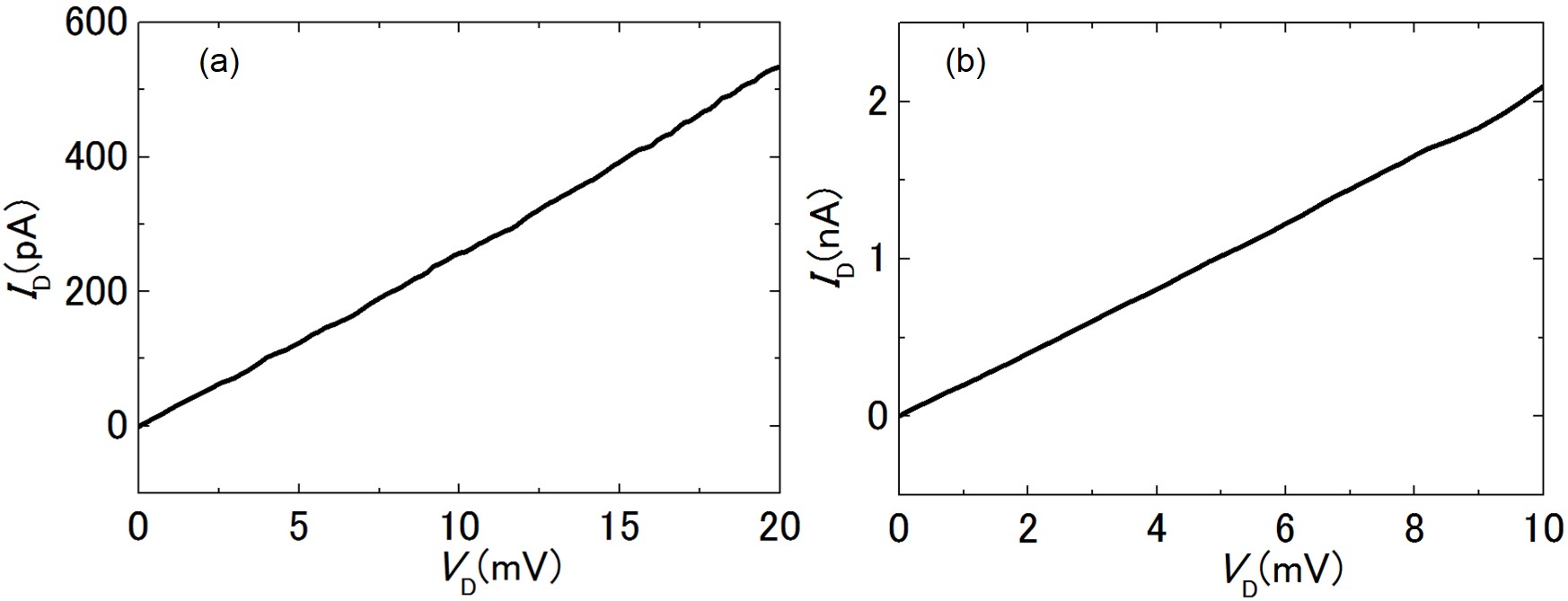


**Figure S2.** *I*_D_–*V*_D_ characteristics of Device A (a) and Device B (b). The Coulomb blockade oscillations shown in Figs. 2 and 3 in the main text were measured at *V*_D_ = 20 mV (Device A) and at *V*_D_ = 5 mV (Device B), respectively. In both devices, *V*_D_ was sufficiently low such that the Coulomb blockade of the dot was not lifted.

**References**

1. Gyakushi, T., Asai, Y., Tsurumaki-Fukuchi, A., Arita, M. & Takahashi, Y. Periodic Coulomb blockade oscillations observed in single-layered Fe nanodot array. *Thin Solid Films* **704,** 138012 (2020).
